# Deformation behavior and properties of severe plastic deformation techniques for bulk materials: A review

**DOI:** 10.1016/j.heliyon.2023.e16700

**Published:** 2023-05-26

**Authors:** Eman M. Zayed, Mostafa Shazly, A. El-Sabbagh, Nahed A. El-Mahallawy

**Affiliations:** a Department of Design and Production Engineering, Faculty of Engineering, Ain-Shams University, Abaseia, Cairo, Egypt; b Mechanical Engineering Department, Faculty of Engineering, The British University in Egypt (BUE), Egypt

**Keywords:** SPD techniques, Metallic bulk samples, SPD characteristics, Industrial SPD approaches

## Abstract

This paper presents a comprehensive review of the successful application of Severe Plastic Deformation (SPD) in producing ultra-fine grained (UFG) and nano-structured crystalline bulk materials. SPD achieves outstanding grain refinement without significantly altering the original dimensions of the workpiece, making it particularly useful for ductile materials that can withstand large strains under high hydrostatic pressure before failure. The study explores the grain refining mechanism during severe plastic deformation and its impact on the microstructure of metals. It also examines the use of SPD in hard to deform brittle materials like tungsten oxide, B_2_O_3_ glasses, and amorphous materials. The paper discusses the advantages and disadvantages of each technique, along with their applications and potential for combining more than one technique. The review is significant because it emphasizes recent progress in process development, which could potentially enable the industrialization of certain SPD techniques for specific applications. This paper fills the gap in the literature by addressing this issue. Overall, the review demonstrates the potential of SPD in metalworking and its application in the development of new UFG materials with improved mechanical properties.

## 1 Introduction

The development of materials with improved mechanical properties is a critical area of research in the field of material science. Severe Plastic Deformation (SPD) has emerged as a promising technique for achieving ultrafine-grained (UFG) materials with exceptional properties. This review paper explores the successful application of SPD in developing UFG and nano-structured crystalline materials. The study discusses various techniques for bulk materials which can be used to achieve UFG materials through SPD.

Some of the common applications of SPD techniques include the aerospace industry, biomedical implants, automotive industry, electronic industry, energy industry, and sports equipment industry. In the aerospace industry, SPD techniques are used to produce ultrafine-grained titanium, aluminum, and magnesium alloys, which have improved strength and fatigue resistance. Biomedical implants require materials with excellent biocompatibility and mechanical properties. SPD techniques can be used to produce ultrafine-grained materials with improved mechanical properties and biocompatibility. For example, ultrafine-grained titanium alloys produced by SPD techniques have shown improved biocompatibility and mechanical properties compared to conventional titanium alloys [1]. In the automotive industry, SPD techniques can be used to produce ultrafine-grained aluminum, magnesium, and steel alloys, which have improved strength and ductility. The electronic industry requires materials with high thermal and electrical conductivity. SPD techniques can be used to produce ultrafine-grained copper, aluminum, and nickel alloys, which have improved thermal and electrical conductivity. The energy industry requires materials with high strength and corrosion resistance. SPD techniques can be used to produce ultrafine-grained stainless steel, nickel-based alloys, and titanium alloys, which have improved strength and corrosion resistance. The sports equipment industry requires lightweight and strong materials to improve performance. SPD techniques can be used to produce ultrafine-grained aluminum alloys and titanium alloys, which have improved strength and fatigue resistance.

Therefore, SPD techniques have a wide range of applications in various industries, and the development of new SPD techniques and optimization of existing techniques can lead to producing materials with enhanced properties [2].

## 2 SPD mechanisms

### 2.1 Severe plastic deformation techniques for bulk samples

#### 2.1.1 High pressure torsion (HPT)

**Fig. 1:**
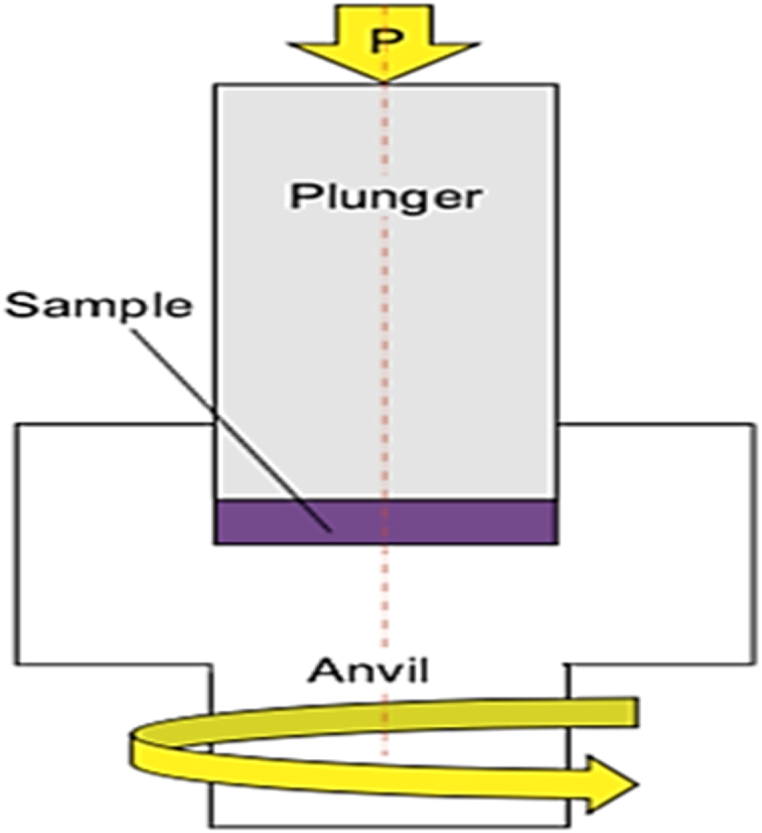
Schematic representation of HPT processing [3].

HPT method which is presented by Ref. [3] as shown in Fig. 1 is a powerful SPD method that imposes high shear strain to the sample. The corresponding strain is determined by Eq. (1) [3]. The HPT method has many advantages as Most SPD techniques impart cyclic strain to the samples, but HPT maintains continuous strain variation [3]. In the HPT process, shear strain could be obtained quite easily. Hard to deform materials can be processed by HPT process because of the high hydrostatic pressure which is difficult to obtain by other SPD methods. Additionally, the change of rotation direction generates SPD, which is common in many other SPD techniques [4].

1 $$\epsilon_{eff}=N\frac{2}{\sqrt{3}}\frac{\pi r}{t}$$

r-distance from center

t-material thickness.

N- Number of revolutions [3].

**Fig. 2:**
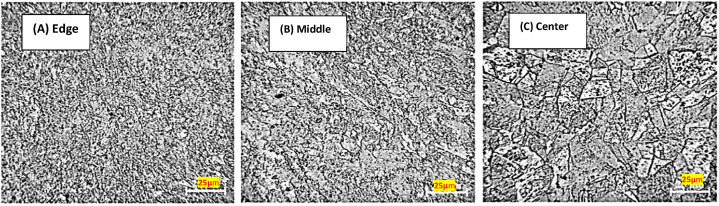
Optical micrographs of HPT processed sample grain size [7].

However, the limitations for this technique are the processing of only small coin shaped samples which make this method limited for research purposes due to size constrains. Also, the non-uniform deformation which could be improved by considering the thickness (h) to the diameter (d) ratio of the sample, that relies on the material of the sample and should not exceed a specific value [5]. Fig. 2a-c illustrates the grain structure evolution as a function of radial direction in HPT processed samples. Three sites (edge, middle, and center) are chosen to demonstrate grain size trends, which reflect the inverse relationship between grain size and strain values in the material, as previously investigated and published in the literature [6,7].

#### 2.1.2 High pressure extrusion (HPTE)

**Fig. 3:**
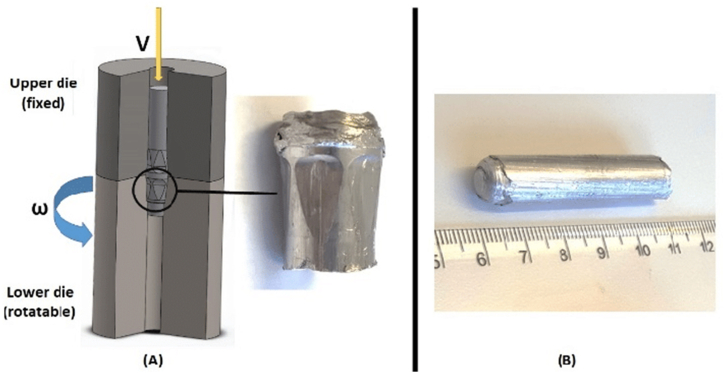
Schematic figure of the HPTE method with a sample during processing [10].

**Fig. 4:**
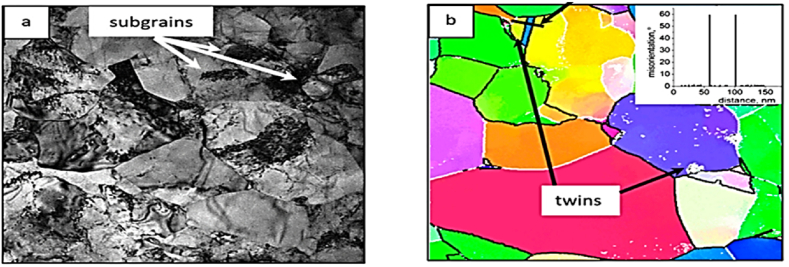
The microstructure of copper processed by HPTE in the central area of the longitudinal section [9].

HPTE technique is a new SPD method for metals and alloys that was developed to enable the production of lengthy ultrafine-grained products with high dislocation density by applying high shear deformation in the active zone, overcoming previous size limitations. The process involves extruding a cylindrical specimen through coaxial rotating containers, with shear deformation occurring at the point of contact as shown schematically in Fig. 3 [8]. Copper specimens were experimentally and analytically processed using HPTE. Fig. 4a shows TEM bright field (BF) images where no precipitates were observed in the grains after HPTE [9], where the grain morphology is equiaxed as shown in Fig. 4b and average grain size is relatively smaller than those in the middle area. The second-phase particle volume fraction is lower than the core area, according to the SEM-BSE pictures.

#### 2.1.3 Equal-channel angular pressing (ECAP)

**Fig. 5:**
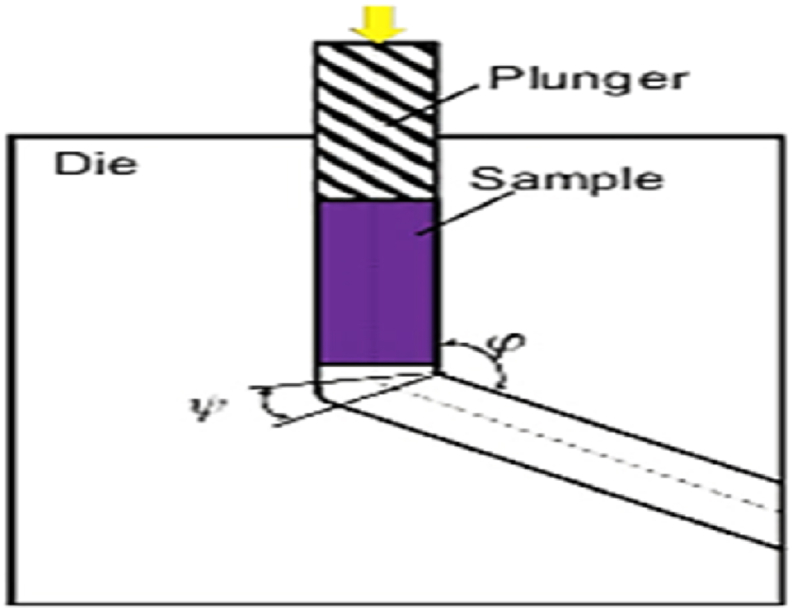
Schematic representation of ECAP process for bulk samples processing [11].

(ECAP) is known as equal-channel angular extrusion. Fig. 5 shows the schematic of a traditional ECAP process. The die consists of two equal channels crossed at an angle φ. This angle is typically chosen to be 90°, however other angles of 60° or 120° are also used. For ease of installation into the inlet channel, the specimen is machined smaller than the channel. Then the sample is pressed into the deformation zone with a plunger. Hence, the specimen is exposed to a large shear plastic strain [11]. The corresponding strain after N passes can be estimated by Eq. (2) [12].

2 $$\epsilon =\frac{2N}{\surd 3}\cot\left(\frac{\varphi }{2}\right)$$

**Fig. 6:**
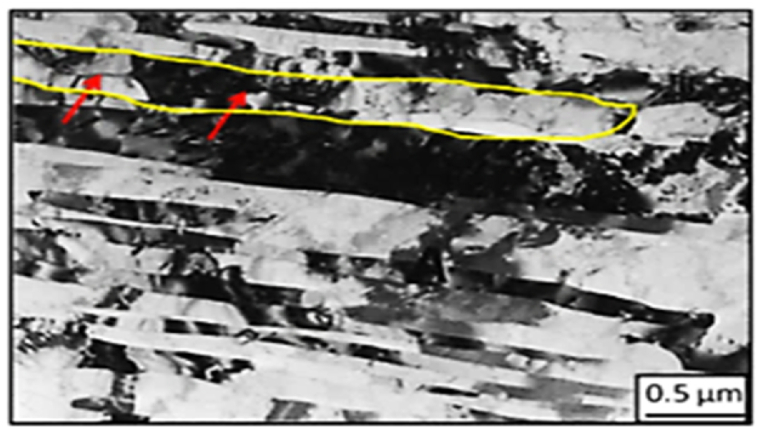
TEM image of ECAPed titanium sample shows twinning and the dislocation walls [13].

Fig. 6 displays the grain refinement mechanism in a pure Titanium sample after one pass of ECAP, where micro-twin bands that characterized by dislocation walls, are shown with red arrows, creating a lamellar structure. This mechanism is also observed in Face-Centered Cubic (FCC) metals, like copper samples, that are subjected to high strain rate methods such as shock loading, ball milling, and dynamic plastic deformation. In these materials, the dislocation density increases due to the presence of active slip planes, leading to the multiplication and migration of dislocations, refining the microstructure. On the other hand, the twinning mechanism has a significant impact on the plastic deformation of Body-Centered Cubic (BCC) and Hexagonal Close-Packed (HCP) metals, including brittle materials like tungsten oxide and B_2_O_3_ glasses, as these materials have restricted slip systems at room temperature. This mechanism also affects ductile materials that can withstand high hydrostatic pressure before failure. Moreover, SPD induces structural alterations in these materials, leading to improved mechanical properties [13,14]. [12].

**Fig. 7:**
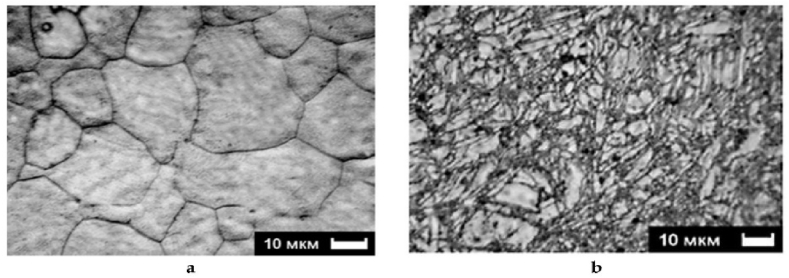
AZ31 microstructure a) as received b) after 2 passes at 200 °C [15].

The microstructure examination reveals typical aspects of grain refinement. Fig. 7 (a) and (b) show SEM images of the AZ31 alloy and after two passes of ECAP at 200 °C. Initially, the grains have equal axis forms with classic triple joint features. After deformation by ECAP, there is grain refinement followed by new high angle grain boundaries being formed along with a huge number of deformation twins [15].

#### 2.1.4 Rotary-die (RD)

**Fig. 8:**
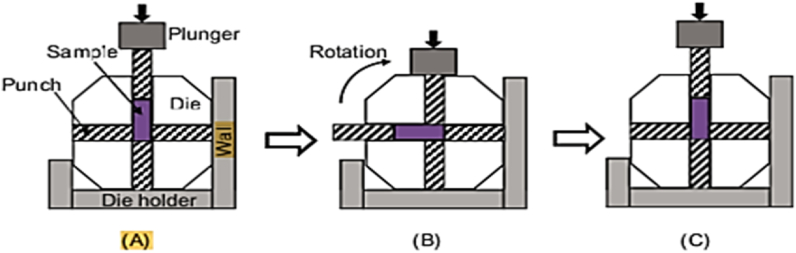
The representation of ECAP rotary-die: (A) original condition, (B) after one pass, and (C) after 90° rotation for the next cycle [16].

Fig. 8 illustrates the ECAP process using rotating dies. A die with two crossed perpendicular channels is used in this operation. The sample is inserted from the top of the die as shown in Fig. 8A, and then pushed laterally by the plunger. The process ends when the plunger reaches the flat surface, and a 90° rotation is applied to the entire die in Fig. 8B. Therefore, the die is prepared for the following cycle without removing the sample, as shown in Fig. 8C. If heating is necessary between two successive cycles, a moving oven heats the entire die set to the specified temperature [16]. However, one of the limitations of this process is that it applies small amount of strain to the sample in a single cycle. As a result, the sample must go through multi passes, that is both time-consuming and challenging.

**Fig. 9:**
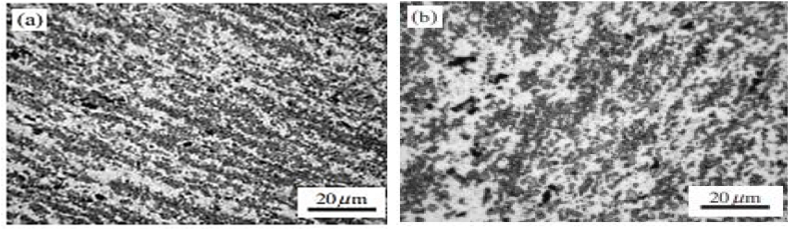
RDECAP micrographs of Al–Si alloys (a) 8 times, (b) 16 times at temperature of 300 °C [17].

Fig. 9 displays the microstructures of Aluminum silicon alloys that have undergone RD-ECAP processing, specifically after 8 and 16 cycles at 300 °C, following T6 treatment. The microstructures illustrate that the distribution of silicon particles and intermetallic compounds within the Aluminum matrix is more even in comparison to the as-cast alloy. Furthermore, it was observed that the uniform distribution of particles increased as the number of RD-ECAP processing cycles increased from 8 to 16 at 300 °C as shown in Fig. 9 a and b respectively [17].

#### 2.1.5 Side extrusion (SE)

**Fig. 10:**
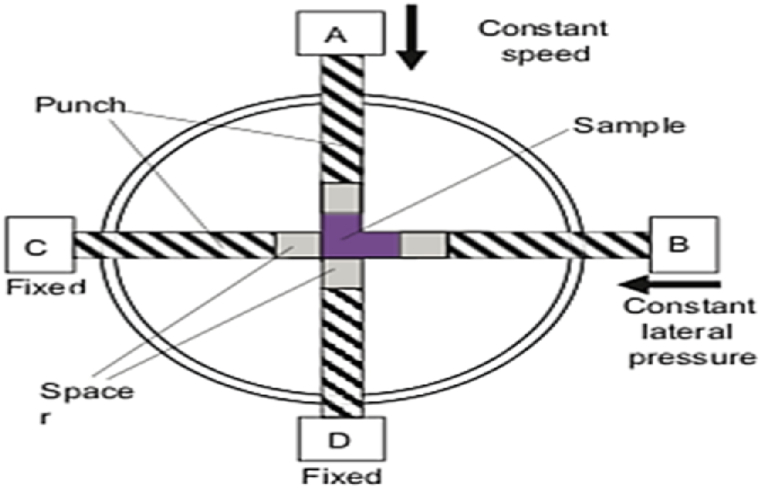
Illustration of the side extrusion ECAP method [18].

The principle of the SE method is identical to the ECAP technique performed with the rotary die, and both approaches use route (A) to impose strain on the sample without rotating. This approach employs movable punches that can apply sufficient force to the sample, as shown in Fig. 10. The electrohydraulic system drives and controls these punches to move a specific distance. Side extrusion is used to move the specimen from channel A to channel B, while punches C and D remain fixed. The control system moves punch A at a constant speed, while punch B delivers a specified side pressure on the sample during deformation. The function of punch A is switched with punch B in the next cycle, and the procedure is repeated. This avoids removing the sample from the die in the subsequent cycle. However, the primary limitation of this method is the high cost and complexity of the control systems [18].

#### 2.1.6 Torsional-ECAP (T-ECAP)

**Fig. 11:**
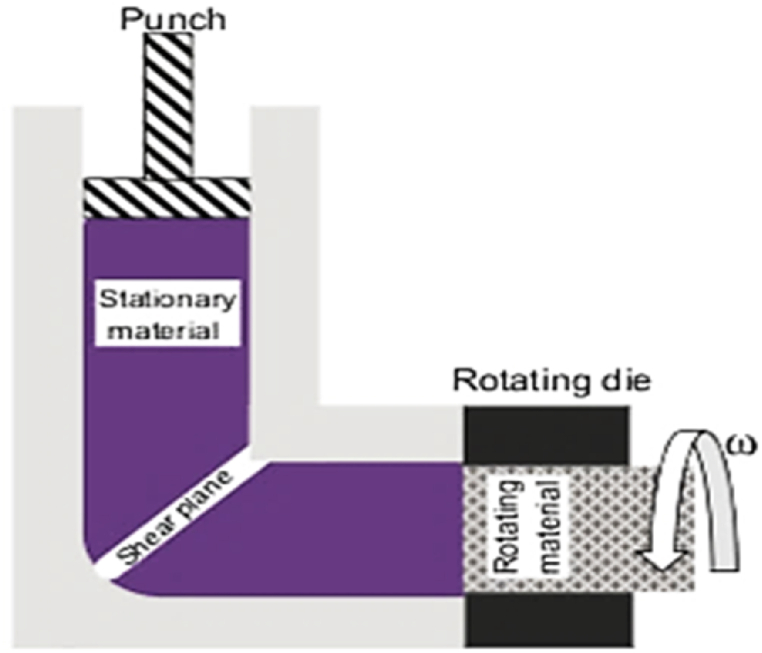
Torsional ECAP setup [20].

The authors utilized the ECAP technique to develop new methods for consolidating aluminum and copper powders. They combined two techniques, ECAP and torsional extrusion (TE), to effectively consolidate pure aluminum powders into fully dense bulk material using a specially designed ECAP set-up. This process is called T-ECAP and involves rotating the output channel during the ECAP procedure, resulting in high-density specimens with fine grains at lower deformation temperatures. The extrusion during the final deformation stage also increases the strain imposed on the sample compared to traditional ECAP. Schematic representation of the T-ECAP is presented in Fig. 11 [19,20].

#### 2.1.7 Back pressure ECAP

**Fig. 12:**
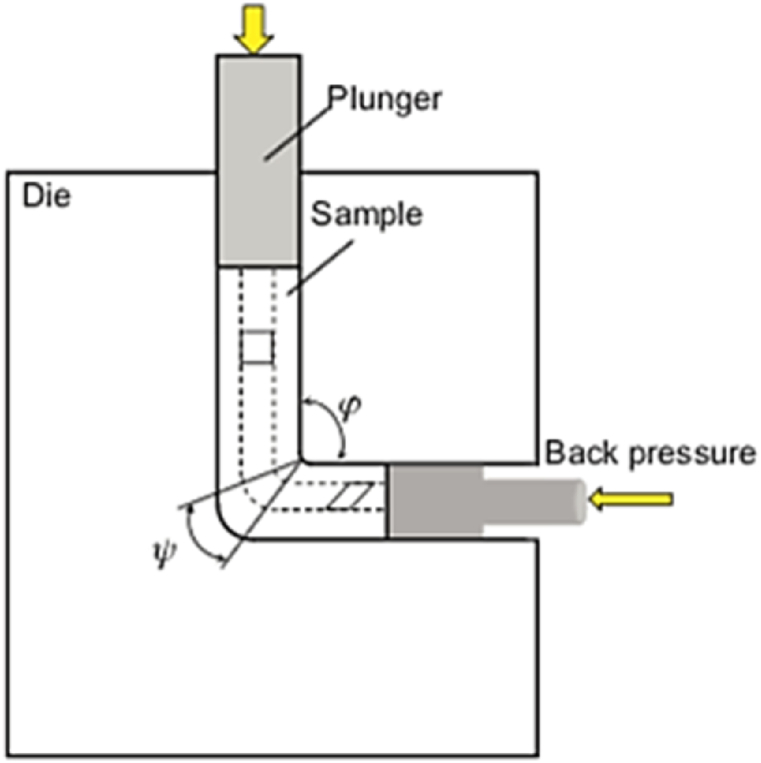
Schematic of ECAP with back pressure technique [21].

**Fig. 13:**
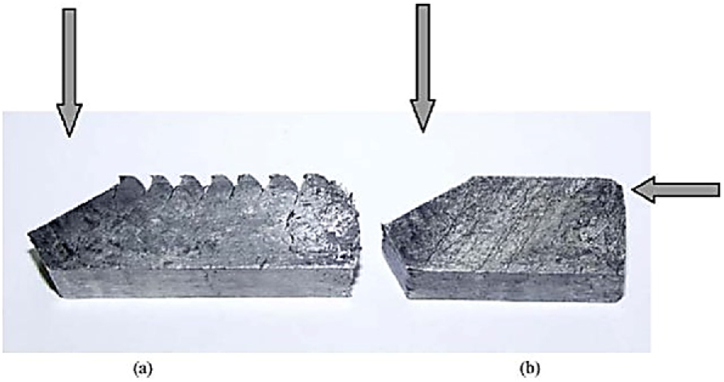
ECAP-processed compacted particles of magnesium alloy with (a) no back-pressure and (b) 60 MPa back-pressure [24].

According to prior research [21], the non-uniformity of strain and stress in samples processed by conventional ECAP depends on the die geometry and material type. This non-uniformity can result in increased surface cracks, leading to fracture during the ECAP process. To address this issue, various approaches have been implemented to improve the uniformity of deformation, such as pre-straining and back pressure utilization (as depicted in Fig. 12) [22]. In a study involving the production of a solid tubular sample from magnesium particles using ECAP under back-pressure (as shown in Fig. 13b, the corner gap shown in Fig. 13a between the die and the sample was eliminated with the application of back pressure. It was discovered that shear deformation with forced hydrostatic pressure on the sample resulted in a homogeneous distribution of stress and strain in the final product, delaying sample fracturing during the ECAP process by increasing compressive hydrostatic stresses [21,23].

#### 2.1.8 Expansion ECAP

**Fig. 14:**
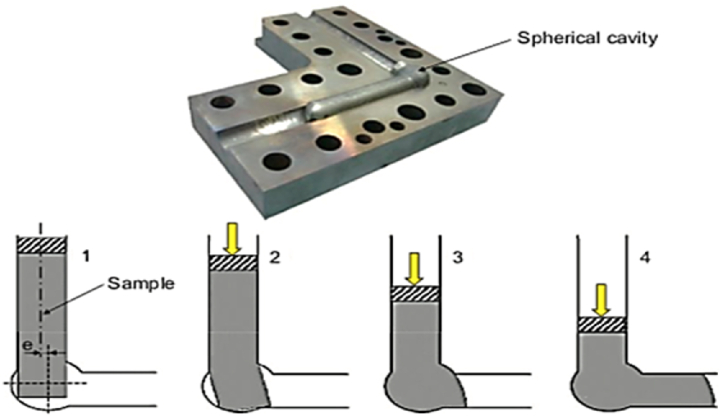
Illustration of Exp-ECAP method, the half of the die containing the deformed material and a schematic of four stages in a cycle [21].

The schematic of the Expansion ECAP technique and the output product prior to separating from the die is shown in Fig. 14 (1–4). The difference between this technique and traditional ECAP is the addition of a spherical cavity at the junction of the input and output channels, which functions as the back pressure behind the sample. This modification has advantages such as imposing bigger stresses and boosting uniformity. The equivalent strain applied during the expansion and extrusion steps is represented in Eq. (3) which describes the relationship between the equivalent strain of a material (ϵ), when it is subjected to a force or load, and various factors that contribute to this strain. These factors include the material's initial length (l), the number of cycles (n), and the ratio of its final diameter to its initial diameter D_1_ and D_0_ respectively as follows:

3 $$\epsilon =4\;\ln\left(\frac{D_{1}}{D_{0}}\right)$$

in this process, better grain refinement will be achieved for the same number of cycles as the total plastic strain is more than the conventional ECAP [21]. [21].

#### 2.1.9 ECAP with parallel channels

**Fig. 15:**
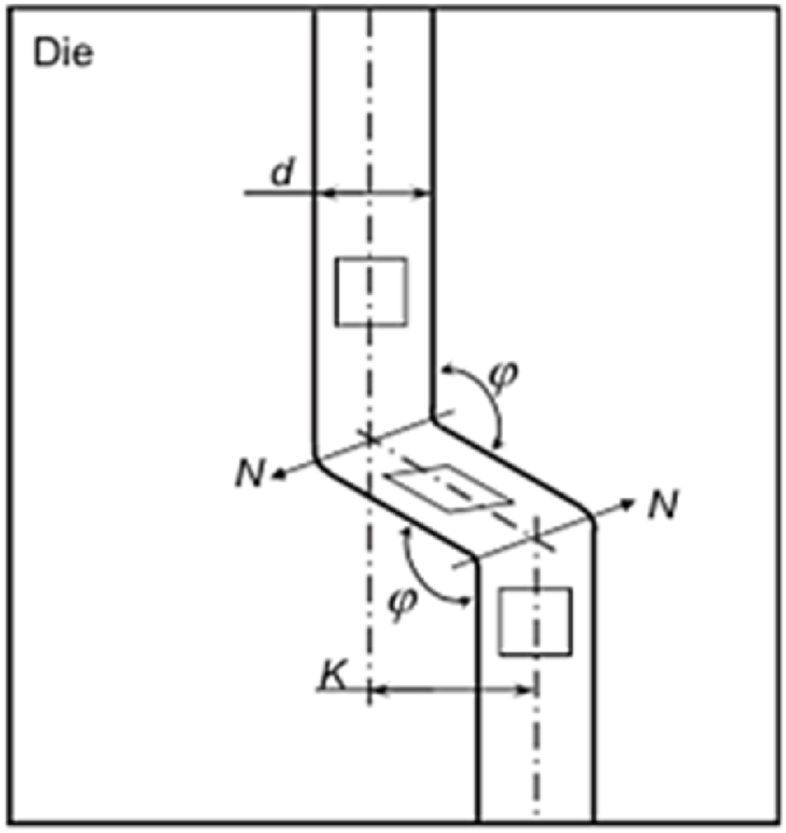
ECAP with parallel channels die design, where N is the direction of shear planes and K is the displacement between the two channels [25].

The modified design of ECAP technique is illustrated in Fig. 15. The inlet and exit channels are parallel with an angle of φ. During ECAP, a sample is pushed between two intersecting tubes with the same cross section, and the material is exposed to severe plastic strain by simple shear.

Shear stress is imposed in the direction of N in two shear planes. By assuming frictionless conditions, Eq. (4) is used to derive the equivalent shear strain (γ) in the deformed sample [26].

4 $$\gamma =2\;\cot\frac{\left(\varphi +\psi \right)}{2}+\psi \;cosec\;\frac{\left(\varphi +\psi \right)}{2}$$

also, the effective strain magnitude (ε) after N passes is calculated by the following formula in Eq. 5

5 $$\varepsilon_{eq}=\frac{N}{3^{1/2}}\left[2\;\cot\frac{\left(\varphi +\psi \right)}{2}+\psi \;cosec\;\frac{\left(\varphi +\psi \right)}{2}\right]$$

This technique has been effectively used for up to four cycles on copper and titanium. Microstructural investigations reveal that the refined grains in these samples are identical to the ultrafine grain structure obtained after eight cycles of ECAP techniques.

#### 2.1.10 Different die designs of ECAP

One of the core drawbacks of traditional ECAP is consuming a lot of energy to overcome the friction between the die and the sample which results in non-uniform mechanical characteristics, poor surface finish, and restricts the maximum length of the sample. Hence, reducing frictional forces is very critical to overcome buckling and yielding of the punches that lead to problems during manufacturing large samples. As a result, various attempts have already been implemented and reported by Ref. [27] to minimize the frictional forces associated with the ECAP process.

**Fig. 16:**
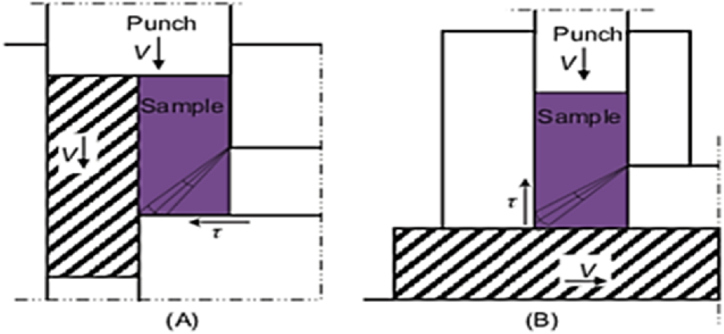
The ECAP principle with adjustable die walls (shown shaded): (A) in the inlet channel and (B) in the exit channel [28].

Two dies with movable walls are designed to reduce the frictional forces as shown in Fig. 16. The first type illustrated by the shaded area in Fig. 16 (A) has a movable inlet channel, however Fig. 16 (B) has a movable wall at the exit channel. The drawback of the movable components is the material extrusion between the separate components. In addition, manufacturing of these dies must be established with minimum intersection point between components to eliminate extra material extrusions and flow of material at high pressure [28]. An alternative technique is proposed to use dies made of solid steel in the ECAP process to prevent issues related splinters of material between the separate parts of the die during the extrusion process.

**Fig. 17:**
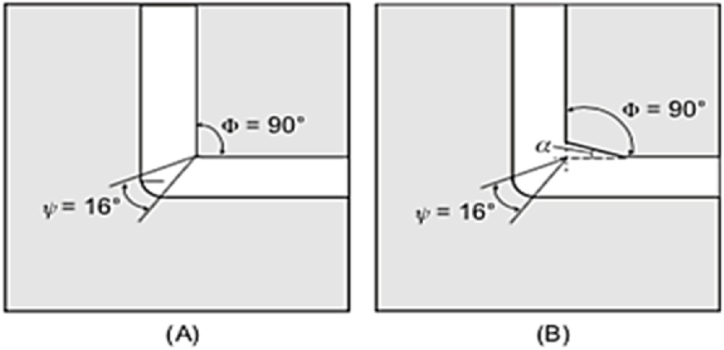
Different configurations of ECAP die: (A) conventional design, (B) modified design [29].

Another modification of the ECAP die can be implemented as shown in Fig. 17 (B) by tilting the die corner with an angle (α) by 2° to 5° degrees compared to the traditional die in Fig. 17A, this can decrease the cracks by increasing the compressive stress. Finite element analysis (FEA) demonstrates the removal of tensile stress and imposing substantial amount of compressive stress on the material by altering the design of the die [29].

#### 2.1.11 Dual equal channel lateral extrusion (DECLE)

**Fig. 18:**
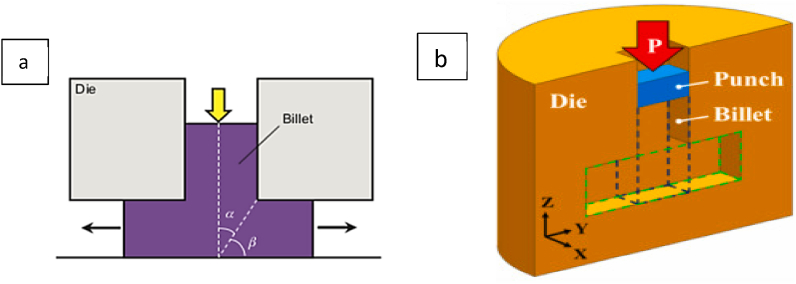
Dual equal-channel lateral extrusion (DECLE) diagram [30].

Fig. 18 (a, b) shows a schematic of the DECLE method where the punch extrudes a specific size of the billet in a vertical T-shaped input channel, which then flows into a horizontal channel with the same inlet dimensions [30].

#### 2.1.12 Channel angular pressing with converging billets

**Fig. 19:**
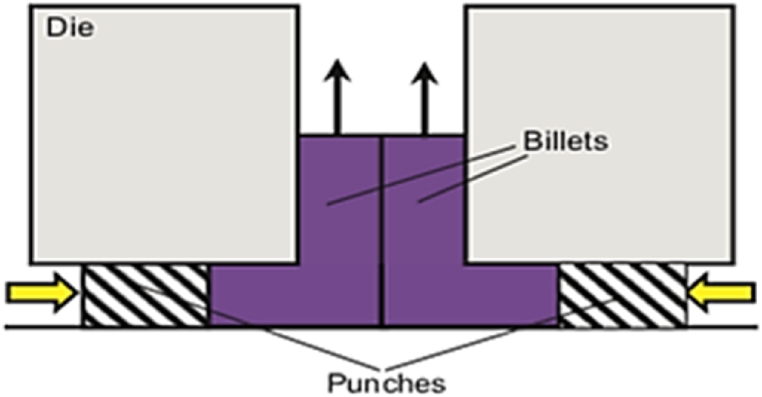
Schematic illustration of channel angular pressing with converging billets [31].

In the technique shown in Fig. 19, two identical punches (square or rectangular) are employed at the input channels to simultaneously press the input billets and combine the two converging billets into a single output channel twice the width of the input channels. This approach reduces processing force and friction during deformation. However, the adhesion of billets causes problems when they need to be separated after processing. The nonuniformity of the initial billets may result in a poor surface quality after separation [31].

#### 2.1.13 Non-equal channel angular pressing [NECAP]

**Fig. 20:**
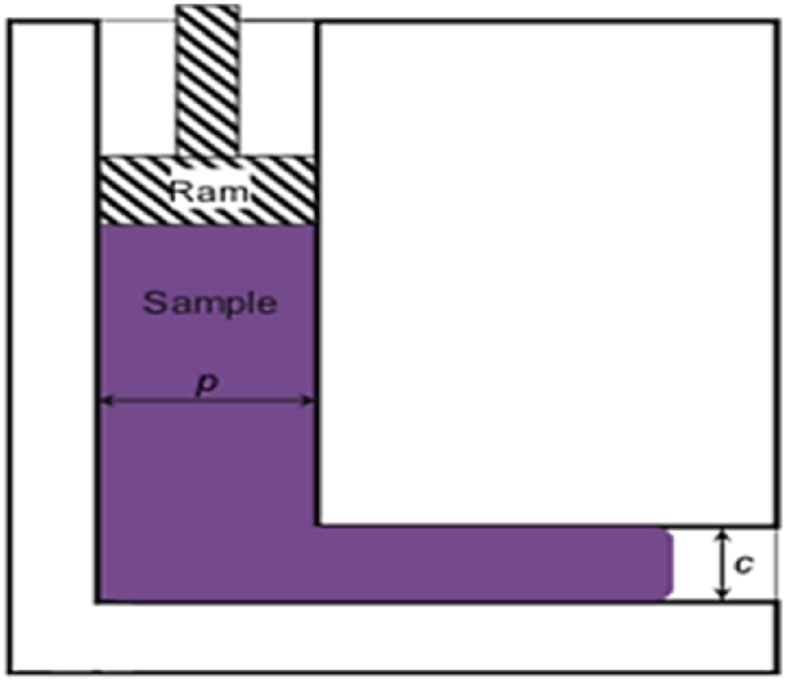
NECAP die Schematic [32].

The NECAP process that is illustrated in Fig. 20 is preferred over the ECAP process because the NECAP process has a larger extrusion ratio, which imposes a higher processing load. As a result, the strain applied on the sample by single pass of NECAP relative to the strain applied by one pass of ECAP is 38% larger [32]. The advantage of the NECAP method in industrial applications is that it produces better grain refinement and uniform microstructure, especially for hard to deform alloys. NECAP can also be used to join two dissimilar metals, like the coextrusion welding method [33].

**Fig. 21:**
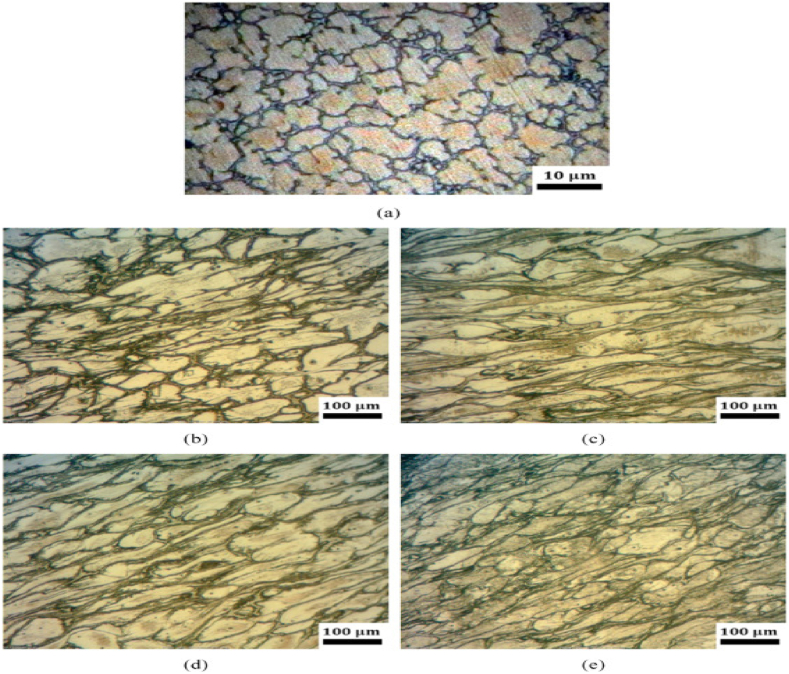
Micrographs of the AZ80 Mg alloy for (a) as cast, and longitudinal direction of the product NECAPed under different process parameters with (a) as received (b) T = 250 °C, v = 1 mm/min, (c) T = 300 °C, v = 1 mm/min, (d) T = 300 °C, v = 2 mm/min, (e) T = 300 °C, v = 4 [32].

Fig. 21a reveals the microstructure images of the as-cast AZ80 Mg alloy with non-deformed grains. Fig. 21b-e illustrates the microstructure of the processed specimen material at various temperatures and velocities. The grain refinement in NECAP process may be higher and faster than those in the ECAP process. The temperature has a noticeable effect on dynamic recrystallization. Because recrystallization is controlled by diffusion, as process temperature increases the rate of diffusion will increase accordingly [32].

#### 2.1.14 Torsion extrusion (TE)

**Fig. 22:**
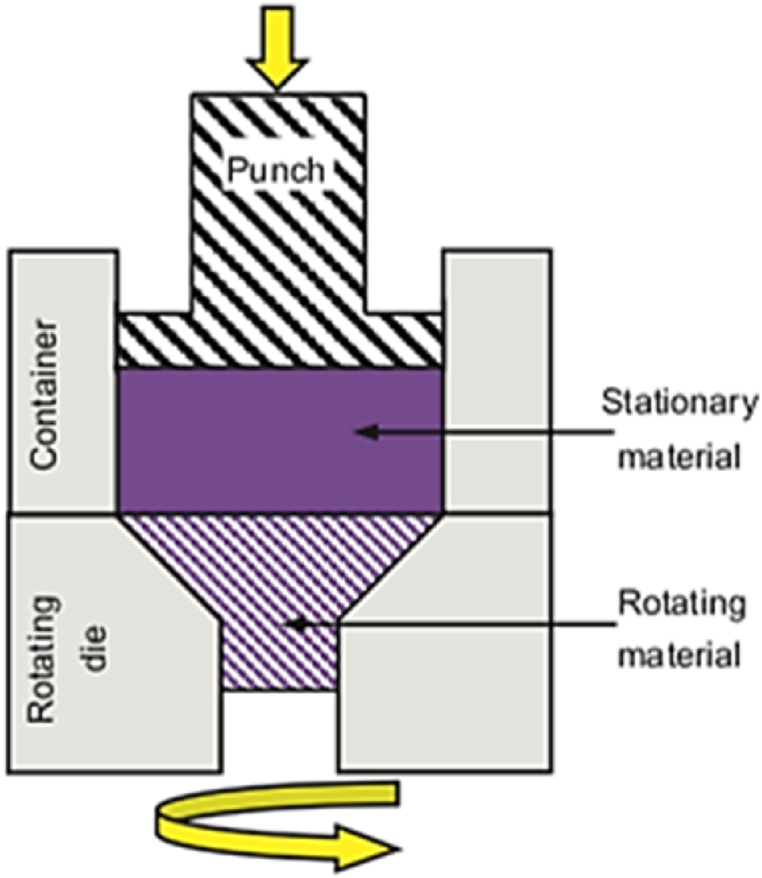
Schematic representation of the torsion extrusion process [34].

Torsion Extrusion (TE) approach, which was introduced in 2006 as a modification to the conventional high pressure torsion technique. Fig. 22 provides a schematic of the TE process, which involves extruding the material through sectional containers that rotate relative to each other to apply high strain on the metal. The shape of the extrusion die may be circular, square, or elliptical. The magnitude of the mean strain can be calculated using Eq. (6).

6 $$\varepsilon =\frac{4\pi RN}{3\sqrt{3}\;H}$$

where R and H are the specimen radius and length respectively, and N is the number of rotations. The sample was more severely strained than that of the traditional extrusion process as reported in prior study [34].

#### 2.1.15 Multiple direct extrusion (MDE)

**Fig. 23:**
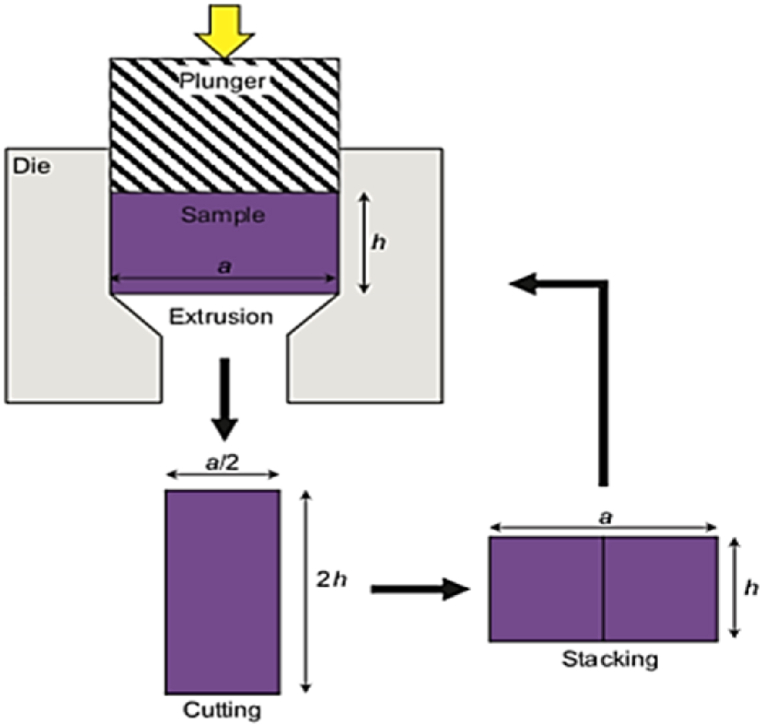
Representation of multiple direct extrusion process [35].

MDE technique was developed based on the direct extrusion method. In this method as shown in Fig (23), the specimen is extruded into a rectangular die with a 50% reduction ratio. After each extrusion cycle, the product is cut at the output ends and joined to obtain the same cubic shape as the original material billet, and the extrusion process is repeated. There are two possible routes for the next cycle: either with no rotation or with a 90-degree rotation around the longitudinal axis of the original billet [35].

**Fig. 24:**
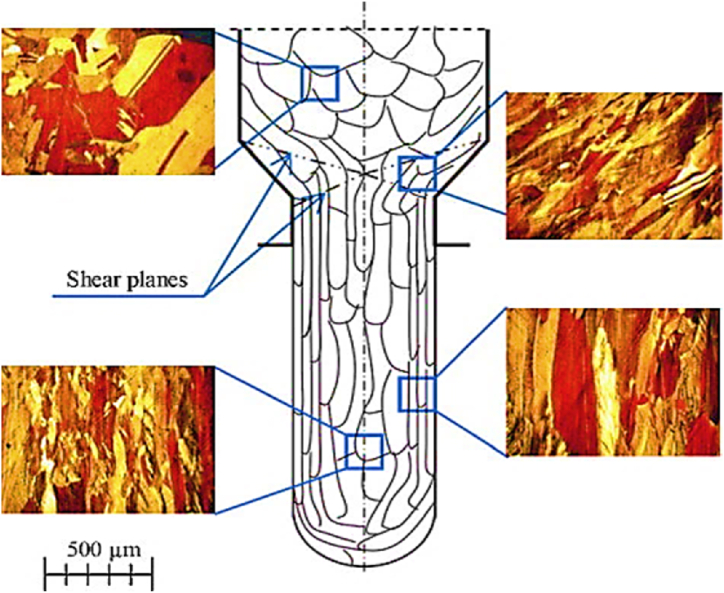
Optical micrographs of processed copper by MDE after one pass [36].

In MDE, when the material passes through the discontinuity zone, the new grains are misoriented along shear planes. The shearing process starts when elongated grains become very thin and tangential stress is applied to the surface of the velocity discontinuity, as shown in Fig. 24 [36].

#### 2.1.16 Accumulated extrusion (AE)

**Fig. 25:**
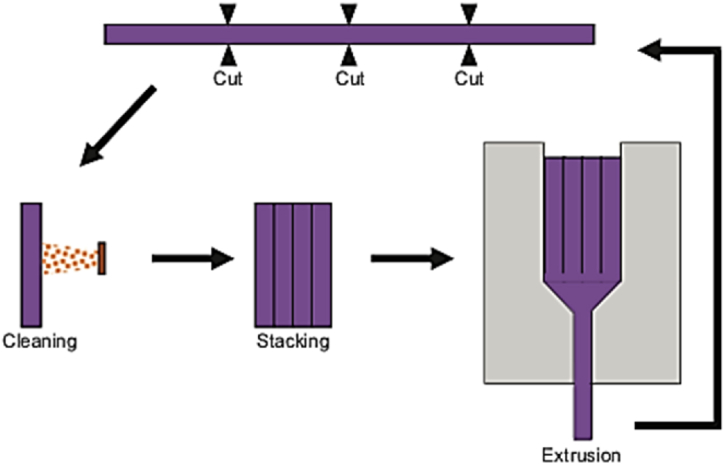
AE process schematic [37].

The (AE) process is illustrated in Fig. 25, which is relatively like MDE process but impose higher strain than the strain obtained in consecutive MDE cycles. In the AE process, the original billet is divided into number of similar sections that are combined and extruded to the final size, and the process is repeated. This approach is implemented to produce multilayer plates. However, the main challenge with both MDE and AE techniques is the bonding and adhesion between the stacked layers [37].

#### 2.1.17 Pure shear extrusion (PSE)

**Fig. 26:**
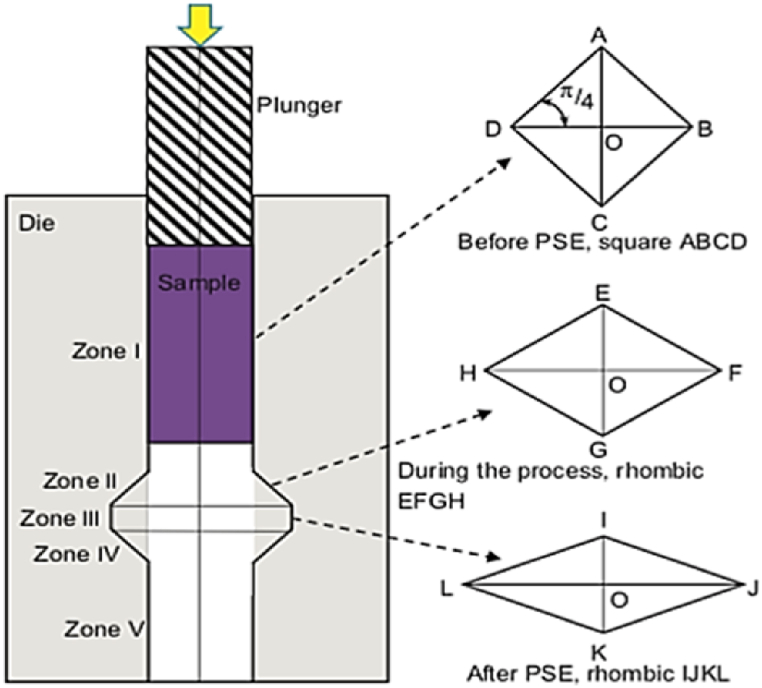
Schematic representation of pure shear extrusion and the variations in the cross-section of the specimen at the half way point of PSE deformation [38].

The PSE process allows combined modes of simple pure shear deformation without repeated work that results in inhomogeneous deformation. Fig. 26 shows, the different deformation zones in the die where the sample is compressed in the middle zone. This modification applies high plastic strain to the sample achieving the desired plastic deformation. PSE can adjust the ratio of pure to simple shear by assuming the shape changes from square to rhombic by PSE deformation [37]. The most significant feature of this process is obtaining relatively large UFG samples without back pressure nor sample rotation [38].

#### 2.1.18 C-shape equal channel reciprocating extrusion (CECRE)

**Fig. 27:**
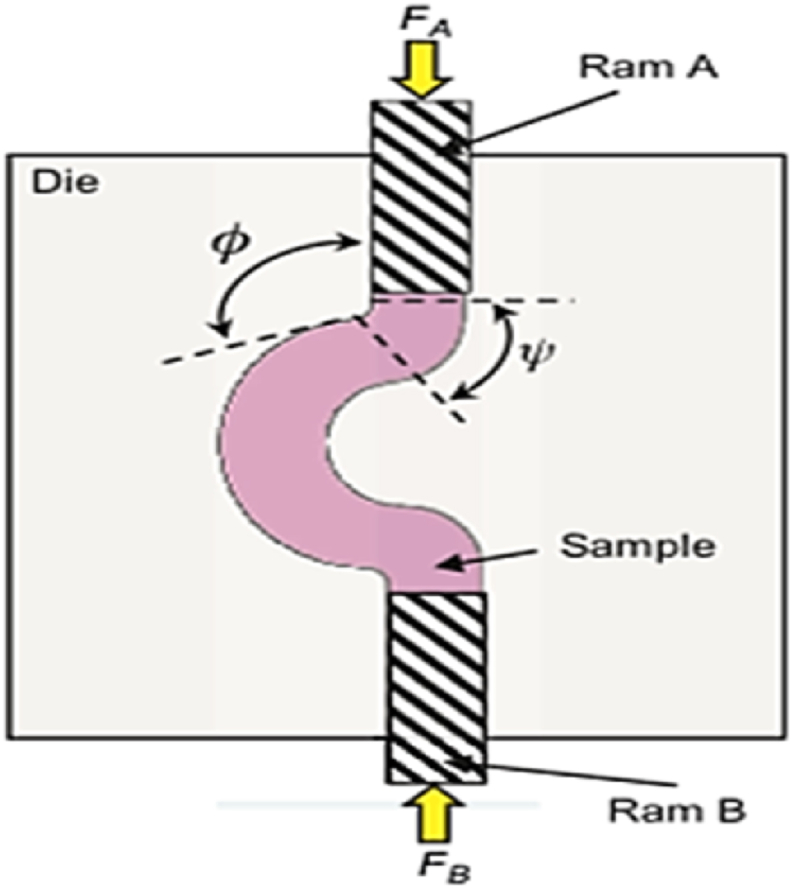
A schematic illustration of the (CECRE) method [39].

The principle of CECRE technique shown in Fig. 27, is to apply shear stress by cyclic deformation. The suggested CECRE method not only exceeds the advantages of the cyclic extrusion compression (CEC), multidirectional forging (MDF), and equal angular channel extrusion (ECAE) processes, but it also overcomes the problems of strain variation between the center and peripheral areas associated with the ECAE, MDF, and CEC processes. Furthermore, it can impose shear strain without affecting the sample size [39].

#### 2.1.19 Twist extrusion (TE)

**Fig. 28:**
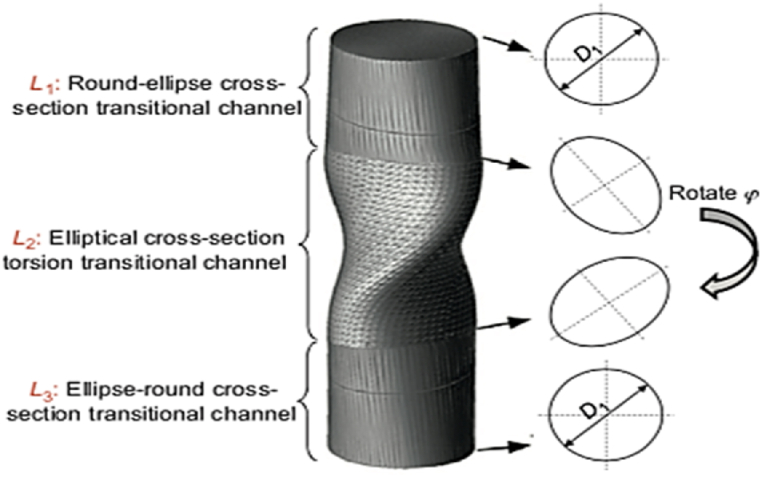
Schematic diagram of ECSEE and PTE [40].

The mechanism of TE shown in Fig. 28 involves extruding the sample through a twist die. The variation of strain from the surface to the center affects the grain size of the sample. The nonsymmetrical products produced by the TE method limits its application to only rectangular samples. Thus, the TE method cannot produce industrial samples due to two factors: buckling and the short travel distance of the plunger. As a result, the two new techniques based on the technique of TE, Elliptical Cross-Section Spiral Equal Channel Extrusion and Planar Twist Extrusion, have been introduced to address the shortcomings of TE. These modifications improve plunger stability, reduce frictional force, and allow producing longer UFG samples [40].

#### 2.1.20 Repetitive upsetting (RU)

**Fig. 29:**
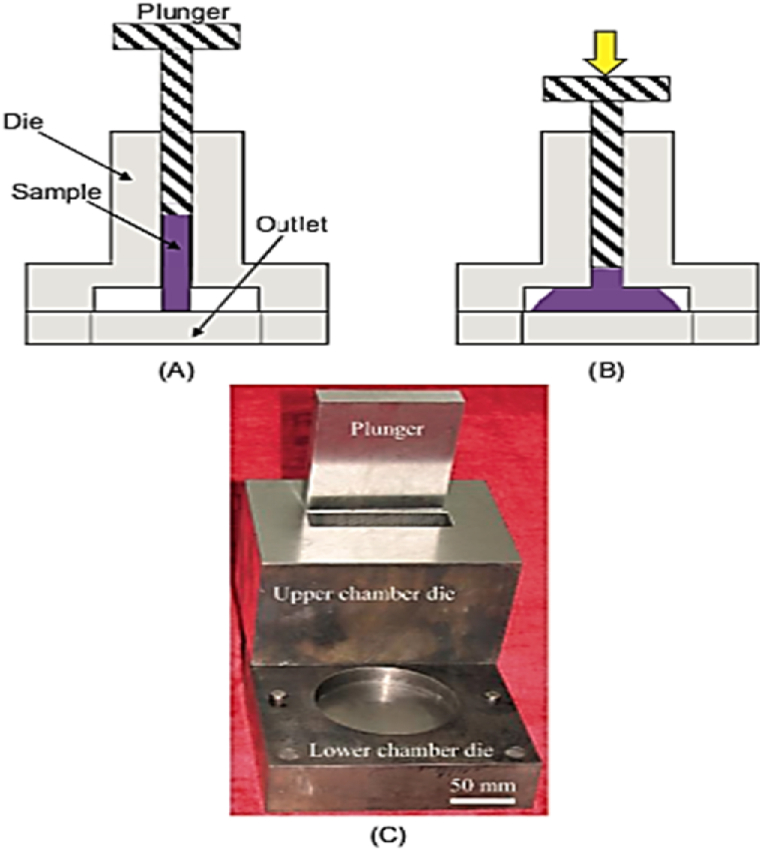
Representation of RU process: (A) at the beginning of the process, (B) throughout the process, and (C) die configuration [41].

**Fig. 30:**
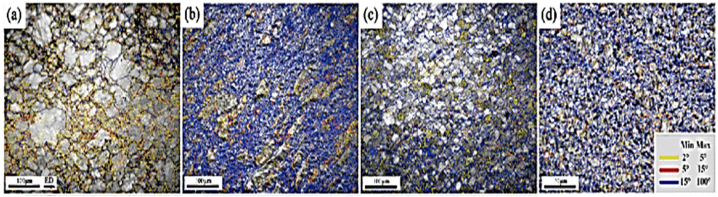
Grain boundaries in processed alloy by RU after several passes [41].

RU process is shown in Fig. 29(A) and (B), applies compressive forces on a disc-shaped sample. The sample is inserted into the die through an upper channel using a plunger as depicted in Fig. 29 (C), and after each pass, it is detached from the bottom of the die and rotated before being reinserted for the following pass [41]. By decreasing the sample thickness, the RU approach can produce industrial-scale thin plates, particularly in hard materials like magnesium alloys [42]. This method can be scaled up for commercial uses. The refined grains shown in Fig. 30 range from 80.45 μm for the original billet Fig. 30 a to finer grain size and more uniform particle distribution in Fig. 30 (b,c) respectively and reaches 4.08 μm after conducting four passes on the sample Fig. 30 (d). The transversal flow of the material in different directions due to increasing deformation amount by RU can be achieved to obtain a homogenous microstructure [43].

#### 2.1.21 Cylinder covered compression

**Fig. 31:**
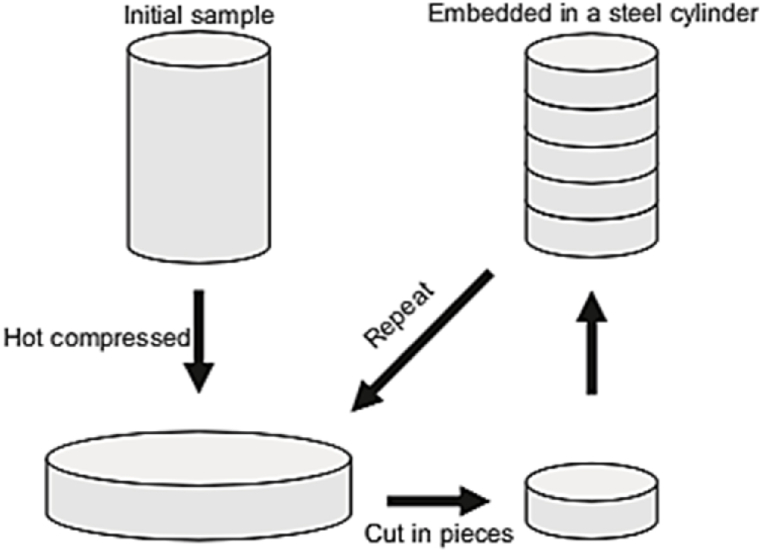
A schematic representation of the CCC technique [44].

The approach described in the paragraph was introduced to address the difficulty of producing ultrafine-grained (UFG) material without cracks in hard deformable alloys like spheroidal cast iron. The method involves several steps, as shown in Fig. 31, where the steel cylinder specimens are hot compressed, cut into equal parts, machining out the surface layers, stacking them, implanting them in a cylindrical die, and then hot compressing them again. This technique has been applied successfully to spheroidal cast iron, resulting in a 99.2% reduction in height [44].

#### 2.1.22 Repetitive upsetting and extrusion (RUE)

**Fig. 32:**
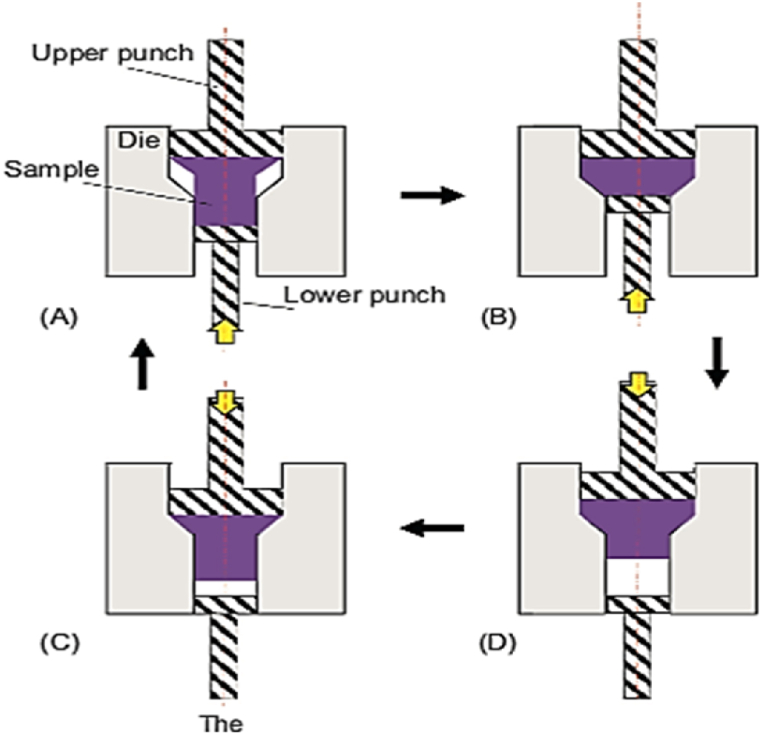
A schematic illustration of RUE method [45].

The mechanism of RUE is represented in Fig. 32, where a circular rod is subjected to upsetting followed by an extrusion process. The operations of upsetting and extrusion are repeated until completing the required number of cycles. REU has various advantages such as imposing high strain per cycle, resulting in more effective grain refinement without applying extra machining of the specimens. Furthermore, warm deformation below the recrystallization temperature could be simply done before the upsetting path to improve the workability of hard materials. The main disadvantage of this technique is that during the upsetting stage, the outer surface is subjected to tensile stresses that cause cracks and reduce the workability of the material [45].

#### 2.1.23 Cyclic extrusion and compression (CEC)

**Fig. 33:**
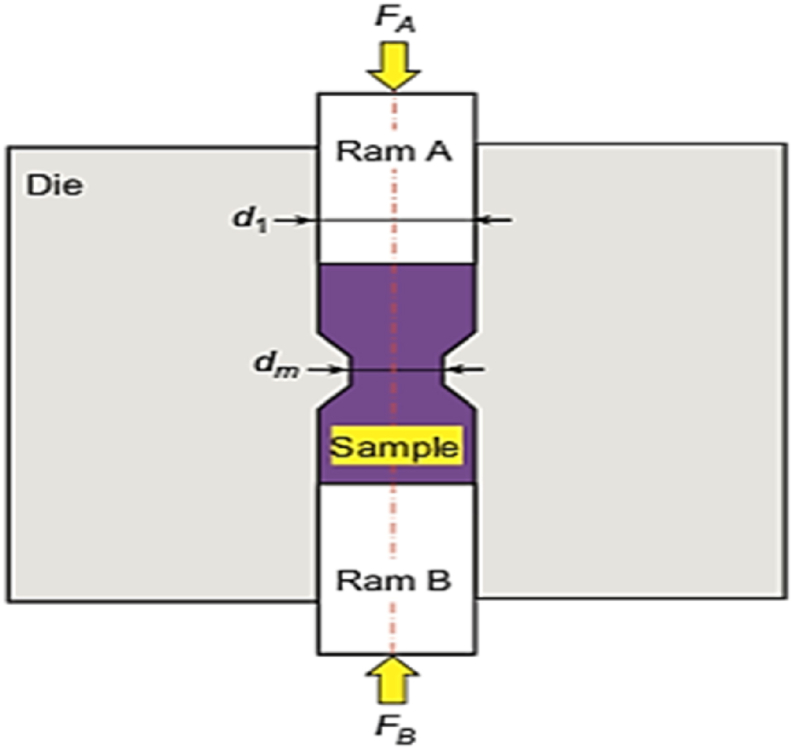
Configuration of the CEC die [46].

CEC method is shown in Fig. 33, where the specimen is pushed into the die to the reduced diameter channel by ram A with a smaller diameter, while a back-extrusion force (F_B_) is applied on the extruded material to restore its initial dimensions. Although CEC is a successful approach for processing hard metals due to the compression state of stress, some materials may flow into the gaps increasing process load and stress. Another constraint of the CEC method which is the limitation of the processed specimen length due to the high friction, which raises the processing load and leads to buckling or yielding of the punch or ram [46,47].

#### 2.1.24 Cyclic expansion and extrusion (CEE)

**Fig. 34:**
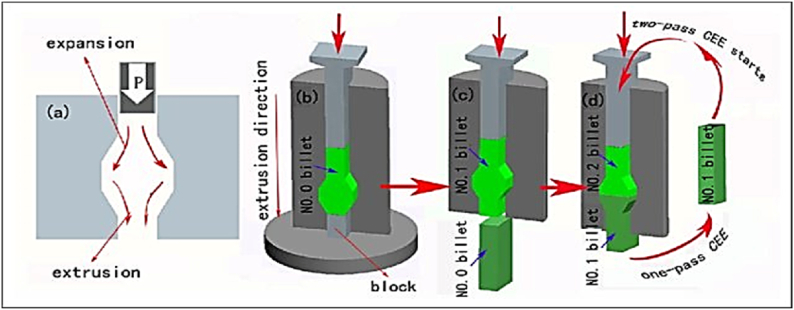
Schematic representation of the CEE stages [48].

**Fig. 35:**
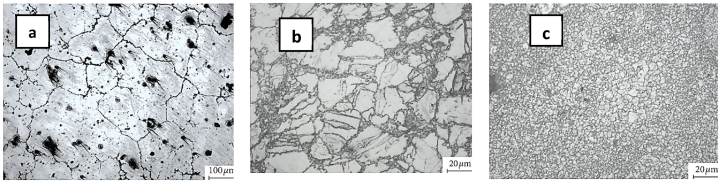
Optical microstructure images of: (a) the annealed sample; (b) one CEE pass; (c) two-passes CEE [49].

The steps involved in the CEE process are illustrated in Fig. 34. First, the primary punch blocks the output channel, resulting in a radial flow of material that fills the barrel zone Fig. 34 (a) and (b). The subsequent extrusion process starts by pushing the upper punch after removing the primary punch, providing the necessary back pressure for material expansion Fig. 34 (c). The process can be repeated without removing the sample from the die if more passes are required. Additionally, the entire die is rotated by 180° Fig. 34 (d). This method can achieve strain values up to 4 and can be used for any number of passes. The microstructure obtained for the annealed sample using the CEE process is shown in Fig. 35a. The optical microstructure images after conducting one and two passes respectively are shown in Fig. 35 (b, c). This approach offers the advantage of producing samples for a specific number of passes without removing them from the die until all passes are completed. Furthermore, unlike CEC, no external back-pressure system is required, as the force needed to extrude the material generates a substantial amount of back pressure for expansion [48,49].

#### 2.1.25 Accumulative back extrusion (ABE)

**Fig. 36:**
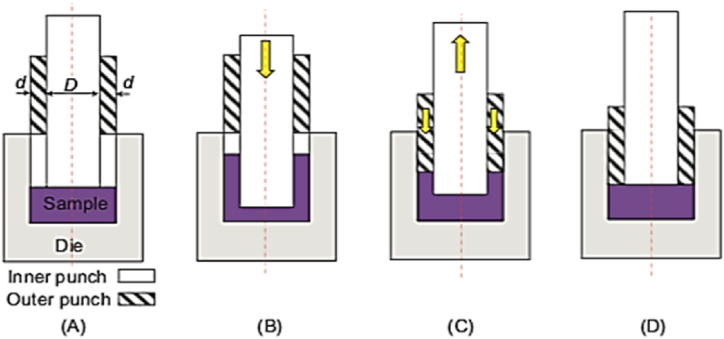
ABE process flow diagram: (A) at the beginning of the cycle, (B) back extrusion, (C) back compression, and (D) end of the cycle [50].

The (ABE) technique is used to produce tubular samples of various sizes. The schematic of ABE technique is shown in Fig. 36A, where the specimen is back-extruded in the space left by the inner punch Fig. 36 B, then compressed back by the outer punch Fig. 36C and the cycle is completed Fig. 36D.This approach can handle large-diameter samples however, cannot produce long samples with uniform microstructures and mechanical properties [50].

**Fig. 37:**
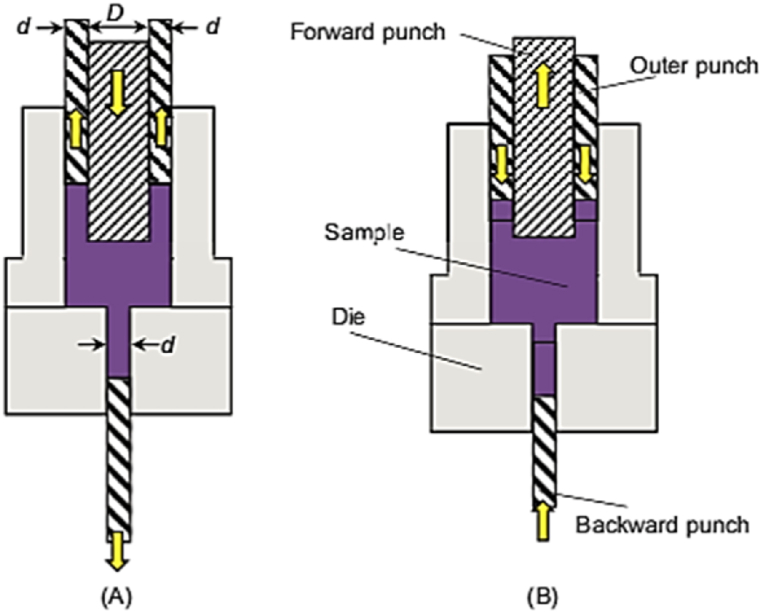
CFBE method: (A) first half-cycle, (B) second half-cycle [50].

Cyclic forward and backward extrusion (CFBE) method is used to process UFG bulk materials at very high strains [50]. As shown in Fig. 37A, the CFBE process is carried out using a dual punch setup consisting of a forward and backward extrusion followed by back-pressure process in Fig. 37B.

#### 2.1.26 Accumulative compression bonding (ACB)

**Fig. 38:**
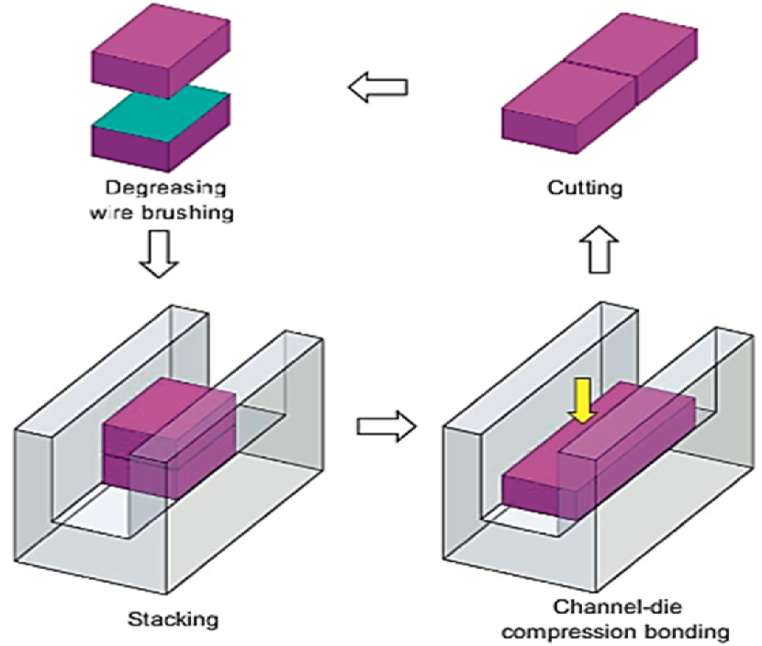
A schematic representation of (ACCB) [51].

The ACB process produces bulk nanostructured metals, as shown in Fig. 38. The specimen is compressed in a channel die causing plane state of strain compression bonding, which is called ideal rolling deformation, where the sample is reduced in thickness and increased in length in the channel die without any lateral spreading. To obtain the original thickness, the sample is wire brushed and degreased prior to compression and cut into two halves, then stacked. In the channel die, the stacked billets are compressed by 50% in the following cycle. To manufacture a bulk sample with adequate bonding. (ACB) can be used to produce homogenous UFG as well as high-strength metal matrix composites (MMCs). Without the need to high-capacity rolling mill, this procedure can be accomplished using a channel die attached to a regular pressing machine. Furthermore, it may be applied to large-dimension samples, and the strain in this process can be precisely controlled by adjusting the pressing speed and the amount of deformation [51]. Prior study examined the effects of strain accumulation and annealing on the interfacial microstructure and grain structure (Mg and Al_3_Mg_2_ layers) of Al/Cu/Mg multilayered composite fabricated by accumulative roll bonding (ARB) process. The results indicated that the strain accumulation led to the formation of a homogeneous interfacial microstructure and a refined grain structure. The annealing process further improved the grain structure and eliminated the defects at the interface. Overall, the study suggests that the ARB process followed by strain accumulation and annealing can produce a composite with improved mechanical properties [52].

### 2.2 Combined SPD methods

Some of the previously outlined technologies can be coupled to produce even larger benefits, resulting in improved material characteristics and process efficiency. These combined approaches have the potential to offer larger equivalent strain on the materials while also overcoming some of the restrictions of SPD techniques [53]. Cold rolling and ECAP that followed by HPT, improved the mechanical characteristics. Another study conducted that combine two SPD methods, ECAP and Extrusion, to create UFG materials such pure nickel and Al–Mg alloy.

## 3 Conclusions

There are various techniques for achieving severe plastic deformation to improve their mechanical properties and microstructure of metals. Traditional methods such as rolling, forging, and extrusion have limitations in achieving high strains, while newer methods such as ECAP, HPT, and others can achieve higher strains and produce finer microstructures. However, these methods also have their drawbacks such as high energy consumption, equipment limitations, and difficulty in producing large samples.

To overcome these limitations, researchers have proposed modifications to existing methods such as using multiple passes, changing the die design, and developing new techniques or by combining two or more SPD processes in a consecutive way. These modifications can improve the efficiency of the process, reduce energy consumption, and improve the mechanical properties and microstructure of the material. However, each method has its own advantages and limitations and choosing the appropriate method depends on the material and desired properties. Overall, these advancements in severe plastic deformation techniques have shown great potential for improving the mechanical properties and performance of metals in various applications. The technical aspects of SPD used in this review can aid the researcher in promoting the findings of earlier studies.

## Author contribution statement

All authors listed have significantly contributed to the development and the writing of this article.

## Data availability statement

Data included in article/supplementary material/referenced in article.

## Additional information

No additional information is available for this paper.

## Declaration of competing interest

The authors declare that they have no known competing financial interests or personal relationships that could have appeared to influence the work reported in this paper.
